# Clinical application of regional citrate anticoagulation for continuous renal replacement therapy in children with liver injury

**DOI:** 10.3389/fped.2022.847443

**Published:** 2022-10-11

**Authors:** Fang Hu, Yuelin Sun, Ke Bai, Chengjun Liu

**Affiliations:** ^1^Intensive Care Unit, Children's Hospital of Chongqing Medical University, Ministry of Education Key Laboratory of Children Development and Disorders, China International Science and Technology Cooperation Base of Child Development and Critical Disorders, Chongqing Key Laboratory of Pediatrics, Chongqing, China; ^2^The People's Hospital of Qijiang District, Chongqing, China

**Keywords:** children, liver injury, liver failure, regional citrate anticoagulation, continuous renal replacement therapy

## Abstract

**Background:**

Regional citrate anticoagulation (RCA) is increasingly used for continuous renal replacement therapy (CRRT) in children, but it is rarely used in children with liver injury, especially liver failure (LF). We analyze this issue through the following research.

**Methods:**

We retrospectively analyzed 75 children with liver injury who underwent RCA-CRRT in the Pediatric Intensive Care Unit (PICU) of Children's Hospital of Chongqing Medical University. The patients were divided into the LF group and liver dysfunction (LD) group. The two groups were compared to evaluate the clinical safety and efficacy of RCA-CRRT in children with liver injury and to explore RCA-CRRT management strategies, in terms of the following indicators: the incidence of bleeding, clotting, citrate accumulation (CA), acid–base imbalance, and electrolyte disturbance, as well as filter lifespans, changes in biochemical indicators, and CRRT parameters adjustment.

**Results:**

The total incidence of CA (TCA) and persistent CA (PCA) in the LF group were significantly higher than those in the LD group (38.6 vs. 16.2%, *p* < 0.001; 8.4 vs. 1.5%, *p* < 0.001); and the CA incidence was significantly reduced after adjustment both in the LF (38.6 vs. 8.4%, *p* < 0.001) and LD groups (16.2 vs. 1.5%, *p* < 0.001). The incidence of hypocalcemia was significantly higher in the LF group than in the LD group either before (34.9 vs. 8.8%, *p* < 0.001) or after treatment (12.0 vs. 0%, *p* < 0.001). The speed of the blood and citrate pumps after adjustment was lower than the initial setting values in both the LF and LD groups. The dialysis speed plus replacement speed were higher than the initial settings parameters.

**Conclusion:**

For children undergoing RCA-CRRT, the risks of CA and hypocalcemia are significantly higher in children with liver failure than those with liver dysfunction, but through the proper adjustment of the protocol, RCA-CRRT can still be safely and effectively approached for children with LD and even LF.

## Introduction

The liver is an important human organ that integrates five main functions, i.e., synthesis, metabolism, detoxification, immunity, and secretion. When liver failure occurs, it can cause severe clinical manifestations or even death. For those with liver failure (LF), CRRT is often needed to eliminate the metabolic waste that the liver cannot remove adequately, such as blood ammonia, blood lactic acid, and some inflammatory factors. But for many other critically ill children, even liver dysfunction (LD) ones, CRRT is also required for reasons such as acute kidney injury, severe infection, severe electrolyte disturbance, toxin, or drug-induced intoxication. Since the liver plays an important role in synthesizing blood coagulation factors, children with liver injury usually have coagulation dysfunction, which brings challenges to anticoagulation for CRRT in these patients. RCA was recommended by 2012 KDIGO as the first preferred anticoagulation method for CRRT without contraindications (1). Citrate is metabolized mainly in the liver, so liver failure was once listed as one of the contraindications of RCA. However, with the development of RCA research, there have been more and more reports on the application of RCA in adult patients with LF in recent years (2–6), but only a few in children (7–12). Few studies introduced the detailed RCA-CRRT protocol, especially those after adjustment. In this study, we aimed to retrospectively analyze the clinical data of the children with different degrees of liver injury who underwent RCA-CRRT in our hospital, to evaluate the efficacy and safety of RCA-CRRT in these children, and explore the optimized RCA-CRRT protocol.

## Data and methods

### General data

This study retrospectively analyzed the clinical data of 75 children with liver injury who received RCA-CRRT in the Pediatric Intensive Care Unit of the Children's Hospital of Chongqing Medical University from January 2015 to October 2019. They were divided into LF group and LD group. The diagnostic criteria for LF (13, 14) were in accordance with international and domestic diagnostic criteria for acute LF in children, including: (a) the absence of pre-existing liver disease, (b) severe LD suddenly occurred within 8 weeks of onset, and (c) uncorrectable coagulopathy (after the administration of vitamin K) with prothrombin time (PT) >20s and international normalized ratio (INR) >2.0 in patients without hepatic encephalopathy, or coagulopathy with PT>15s or INR> 1.5 in patients with encephalopathy. Or, children without the underlying chronic liver disease have acute liver injury accompanied by multiple organ systems malfunction, with or without encephalopathy caused by hepatocyte necrosis. LD was defined as those who met the PODIUM criteria (15, 16) for LD but did not meet the diagnostic criteria for LF, including: aspartate aminotransferase (AST) >100 IU/L or alanine aminotransferase (ALT) >100 IU/L or glutamyl transpeptidase (GGT) >100 IU/L or total bilirubin (TB) >5 mg/dl (>85.5 μmol/L) or direct or conjugated bilirubin (DB) >2 mg/dl (>34.2 μmol/L). This study has been reviewed and approved by the Ethics Committee of the Children's Hospital of Chongqing Medical University, with ethical review number: (2020) NLS (Y) No. 131.

### Data collection

Data recorded include: (i) the patient's age, gender, weight, and other general information; (ii) test results of children's liver and kidney function, electrolytes, coagulation, serum ammonia, and serum lactic acid; (iii) incidences of active bleeding, reasons for disconnecting; (iv) the prognosis of the children; (v) filter lifespan for each CRRT treatment; (vi) blood gas analysis every 4–6 h during treatment; (vii) liver function indicators before, during, and after treatment, including TB, DB, AST, ALT, and GGT; (viii) coagulation indicators (including PT and APTT) and total calcium every 12–24 h.

### Initial RCA-CRRT protocol

**Figure 1:**
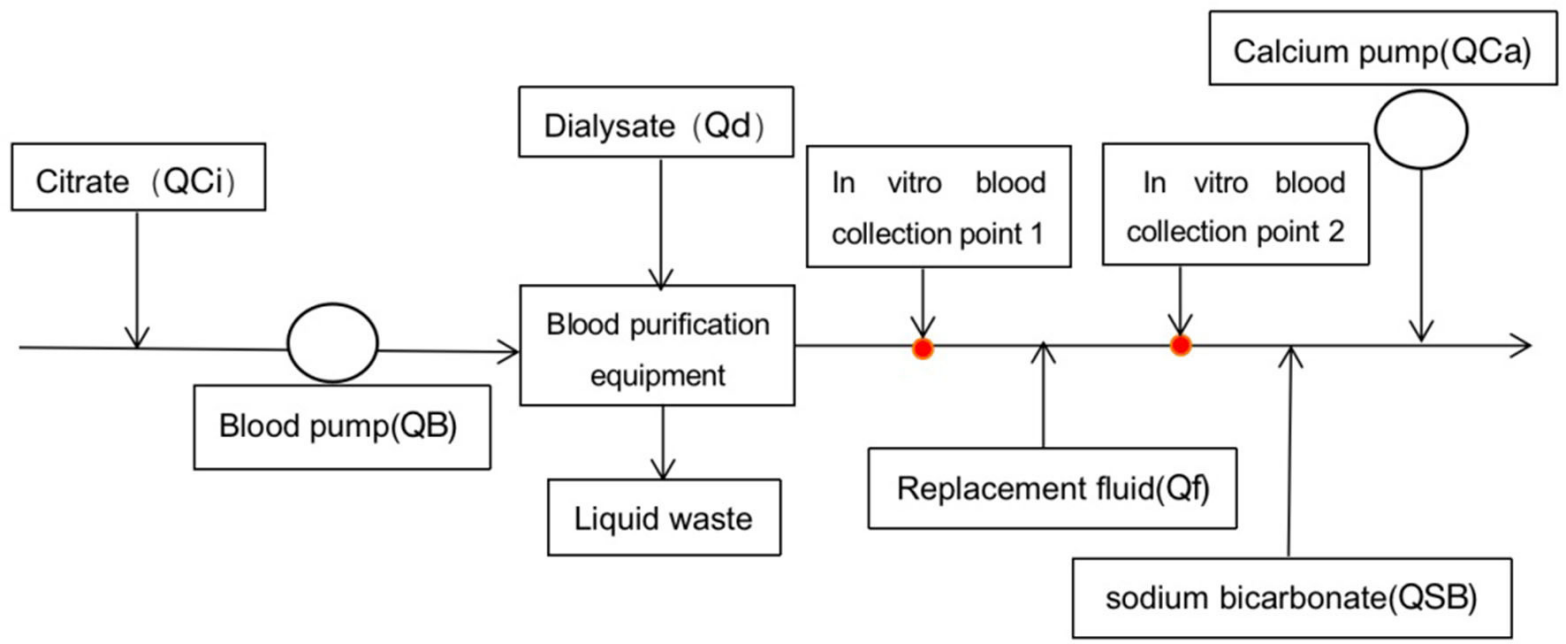
Schematic diagram of hemodiafiltration. Point 1 was the site where the blood sample was collected for the protocol using calcium-free replacement fluid, and point 2 for the calcium-containing protocol.

The blood purification equipment PlasautoΣ was used to perform CRRT. All children underwent continuous diafiltration. The CRRT cycle was flushed with 3 L of heparinized saline solution (40 mg/L), followed by normal saline. If the child had unstable hemodynamics or the volume of the CRRT cycle exceeded one-tenth of the child's total blood volume, the cycle would be flushed with red blood cell suspension. Commercially available HBS (National Medicine Permit No. H20080452; Chengdu Qingshan Likang Pharmaceutical, China) was used as the dialysis and replacement fluids. The citrate sodium anticoagulant (4%; National Medicine Permit No. H20058913; Sichuan Nightingale Biological, China) was pumped into the CRRT cycle through a T-junction connected at the primer of the artery pipeline. Calcium gluconate (10%) and sodium bicarbonate (5%) were pumped into the CRRT cycle through T-junction connected at the end of the vein pipeline, respectively. Initial treatment parameters: blood flow rate (QB) = 5 ml/kg/min, dose of 4% sodium citrate (QCi) = 9 ml/kg/h, dose of 10% calcium gluconate QCa = 0.5 ml/kg/h, dialysate flow rate (Qd) = 25 ml/kg/h, replacement fluid flow rate (Qf) = 35 ml/kg/h, and dose of 5% sodium bicarbonate (QSB) = 0.6 ml/kg/h. Blood gas analysis and electrolytes, *in vitro* and *in vivo*, were measured every half an hour after the start of treatment until the anticoagulation goal was achieved, and then routinely every 4–6 h to maintain target concentrations. First, the flow rate of the 4% sodium citrate was adjusted to maintain the ionized calcium concentration (iCaE), *in vitro*, targeted to 0.2~0.4 mmol/L. Then the infusion rate of 10% calcium gluconate was adjusted to maintain the ionized calcium concentration (iCaI), *in vivo*, targeted to 1.00~1.35 mmol/L. Finally, the concentration of Na^+^ and ${\text{HCO}}_{3}^{-}$ was monitored and adjusted by varying QSB if necessary. After stabilization, the liver and kidney function and the electrolyte were measured every 12~24 h. According to these results, the parameters were appropriately adjusted, and even the calcium-free replacement fluid configured by the nurse was replaced. When using calcium-free replacement fluid, QCi was initially set as 7.5 ml/kg/h. If the ratio of total calcium to ionized calcium >2.5 (T/iCa >2.5) occurred, increasing the flow rate of replacement fluid and (or) dialysate, or even reducing the blood pump speed to reduce the citrate speed and citrate accumulation in the body could be managed. *In vitro* calcium monitoring sites are shown in Figure 1.

### Data interpretation

Filter lifespan is the duration of a session of CRRT. Total citrate accumulation (TCA) is defined as the ratio of total calcium to ionized calcium >2.5 (T/iCa >2.5 occurs once or more during treatment); persistent citrate accumulation (PCA) is defined as if T/iCa >2.5 is present at the end of CRRT. We define hypercalcemia as iCa >1.35 mmol/L, hypocalcemia as iCa < 0.9 mmol/L, hypernatremia as Na^+^ >150 mmol/L, hyponatremia as Na^+^ < 135 mmol/L, severe acidosis as PH < 7.20, severe alkalosis as PH >7.55, normal range of ${\text{HCO}}_{3}^{-}$ as 22–27 mmol/L, normal range of PT as 9.8–13.3 s, normal range of APTT as 25.0–35.4 s, normal range of creatinine as 14–60 μmol/L, normal range of serum ammonia as 9–33 μmol/L, and normal range of serum lactic acid as 0.7–2.1 mmol/L.

### Statistical analysis

The statistical software SPSS 23.0 was used. Counting data were expressed by frequency, and the chi-square test was used. Measurement data conforming to normal distribution were expressed as mean ± standard deviation (*x* ± s), with *t*-test employed for statistical analysis; measurement data of nonnormal distribution were expressed as median (P25, P75), with a non-parametric test (Mann–Whitney *U*-test) employed for statistical analysis. *p* < 0.05 was considered statistically significant.

## Results

### Comparison of general data

There were 42 cases in the LF group (underwent 83 sessions of RCA-CRRT treatments), aged from 4 to 177 months, and 33 cases in the LD group (underwent 68 sessions of RCA-CRRT treatments), aged from 1 day to 192 months. There was no statistically significant difference between the two groups in general data such as age, weight, gender composition, PRISM III Score, and Child-Pugh Score. The general data and causes of LF/LD in the two groups are shown in Table 1.

**Table 1:** General data of children in the LF group and LD group.

	**LF group**	**LD group**	***p*-Value**
**Cases, n**	42	33	/
RCA-CRRT sessions, *n*	83	68	/
Age, months	37 (11.25, 111.25)	49 (9.6, 147)	0.34
Weight, kg	18.84 ± 13.76	24.16 ± 18.73	0.16
Gender ratio, M/F	26/16	24/9	0.25
Child-Pugh Score	12.83 ± 1.90	6.24 ± 0.78	< 0.001
PRISM III Score	20.81 ± 5.65	26.88 ± 6.03	< 0.001
**Causes of LF/LD**			
Toxic hepatitis, *n*	12	3	/
Drug-induced Hepatitis, *n*	5	1	/
Metabolic liver disease, *n*	5	2	/
Congenital biliary tract disease, *n*	3	0	/
Viral hepatitis, *n*	3	0	
Sepsis, *n*	4	14	/
Postoperative congenital heart disease, *n*	1	6	/
Hypoxia, *n*	3	3	/
Other reasons, *n*	6	4	/
Bleeding cases, *n* (%)	4 (9.5%)	3 (9.1%)	0.95
Filter lifespan, h	35.63 ± 17.41	31.94 ± 16.92	0.18
Filter lifespan < 24 h, *n* (%)	21 (25.3%)	22 (32.4%)	0.68
Filters lifespan >48 h, *n* (%)	19 (22.9%)	10 (14.7%)	0.22
**Reasons for disconnecting**			
Clotting, *n* (%)	73	57	0.49
vascular access dysfunction and other issues, *n* (%)	10	11	0.49
Hypocalcemia-associated hypotension sessions, *n* (%)	5(6.0%)	4(5.9%)	1.00
Hypocalcemia-associated convulsions sessions, *n* (%)	2(2.4%)	1(1.5%)	1.00
TCA, *n* (%)	32 (38.6%)	11 (16.2%)	< 0.001
**Correlation between TCA and Child-Pugh Score**			0.739
PCA, *n* (%)	7 (8.4%)	1 (1.5%)	< 0.001
**Correlation between PCA and Child-Pugh Score**			0.891
In hospital mortality	4.8%	6.1%	1.0
28-day mortality, (%)	28.6%	18.2%	0.60

LF, liver failure; LD, liver dysfunction; filter lifespan, filter duration time; TCA, Total citrate accumulation (TCA) is defined as the ratio of total calcium to ionized calcium >2.5 occurs once or more during treatment; PCA, persistent citrate accumulation (PCA) is defined as if T/iCa > 2.5 is present at the end of treatment after adjustment.

### Complications and prognosis

There was no significant difference between the LD and LF groups in 28-day mortality (18.2 vs. 28.6%, *p* = 0.60) and in-hospital mortality (6.1 vs. 4.8%, *p* = 1.0). There were two in-hospital deaths in each of the LF and LD groups, but the cause of death in these four children was not directly related to RCA-CRRT.

The incidence of TCA (38.6 vs. 16.2%, *p* < 0.001) and PCA (8.4 vs. 1.5%, *p* < 0.001) in the LF group was significantly higher than that in the LD group. The incidence of PCA was significantly lower than that of TCA in both the LF (8.4 vs. 38.6%, *p* < 0.001) and LD groups (1.5 vs. 16.2%, *p* < 0.001).

After treatment, the concentration of ionized calcium, *in vivo*, increased significantly both in the LF and LD groups (0.99 ± 0.19 vs. 1.11 ± 0.17, *p* < 0.001; 1.05 ± 0.16 vs. 1.16 ± 0.15, *p* < 0.001). The incidence of hypocalcemia was significantly higher in the LF group than in the LD group either before (34.9 vs. 8.8%, *p* < 0.001) or after treatment (12.0 vs. 0%, *p* < 0.001). The incidence of hypocalcemia was significantly higher before treatment than after treatment either in the LF (34.9 vs. 12.0%, *p* < 0.001) or LD group (8.8 vs. 0%, *p* = 0.01). In terms of hypercalcemia, only the LD group had an increased incidence of hypercalcemia after treatment compared with before treatment (11.8 vs. 1.5%, *p* = 0.02; Table 2).

**Table 2:** Comparison of complications in the intracellular environment between the LF group and LD group.

**Complications (sessions)**	**LF group (*****n*** = **83)**		**LD group (*****n*** = **68)**		***p*-Value**
	**Before treatment**	**After treatment**	**Before treatment**	**After treatment**	
Severe acidosis in sessions (%)	10 (12.0%)	4 (4.8%)	3 (4.4%)	2 (3.2%)	P^F*^= 0.08, P^D*^= 0.50 P^FD#^ = 0.09, P^FD&^ = 0.45
Severe alkalosis in sessions (%)	2 (2.4%)	6 (7.2%)	4 (5.9%)	6 (8.8%)	P^F*^= 0.14, P^D*^= 0.37 P^FD#^ = 0.25, P^FD&^ = 0.47
Hypercalcemia in sessions (%)	0 (0)	3 (3.6%)	1 (1.5%)	8 (11.8%)	P^F*^= 0.12, P^D*^= 0.02 P^FD#^ = 0.45, P^FD&^ = 0.05
Hypocalcemia in sessions (%)	29 (34.9%)	10 (12.0%)	6 (8.8%)	0 (0)	P^F*^ < 0.001, P^D*^= 0.01 P^FD#^ < 0.001, P^FD&^ < 0.001
Hypernatremia in sessions (%)	6 (7.2%)	1 (1.2%)	0 (0)	1 (1.5%)	P^F*^= 0.02, P^D*^= 0.50 P^FD#^ < 0.001, P^FD&^ = 0.70
Hyponatremia in sessions (%)	20 (24.1%)	24 (28.9%)	35 (51.5%)	20 (29.4%)	P^F*^= 0.30, P^D*^= 0.01 P^FD#^ < 0.001, P^FD&^ = 0.53

LF, liver failure; LD, liver dysfunction; P^F*^ is the comparison of the LF group between before and after treatment, P^D*^ is the comparison of the LD group between before and after treatment, P^FD#^ is the comparison before treatment between the LF and LD groups, and P^FD&^ is the comparison after treatment between the LF and LD groups.

During RCA-CRRT, ionized calcium *in vivo* (iCa) monitoring was performed 807 times in the LF group and 666 times in the LD group. There was no significant difference in the incidence of hypotension in RCA-CRRT sessions (6.0 vs. 5.9%, *p* = 1.00) and convulsions (2.4 vs. 1.5%, *p* = 1.00) related to hypocalcemia between the LF and LD groups, as measured by RCA-CRRT sessions (Table 1).

In the LF group, a total of 630 *in vitro* ionized calcium (iCaE) assays were performed with an average iCaE concentration of 0.36 ± 0.09 mmol/L, 421 assays (66.83%) of iCaE between 0.2 and 0.4 mmol/L and 200 assays (31.75%) of iCaE >0.4 mmol/L. In the LD group, a total of 541 iCaE assays were performed with an average iCaE concentration of 0.36 ± 0.08 mmol/L, 363 times (67.10%) of iCaE between 0.2 and 0.4 mmol/L, and 157 times (29.02%) of iCaE >0.4 mmol/L. There was no significant difference between the two groups in the average concentration of iCaE (*p* = 0.06), and no significant difference in the rate reached 0.2–0.4 mmol/L and the rate of iCaE >0.4 mmol/L (*p* = 0.95, *p* = 0.34).

After treatment, the PH (7.38 ± 0.14 vs. 7.45 ± 0.11, *p* = 0.01) and ${\text{HCO}}_{3}^{-}$ (21.40 ± 7.40 mmol/L vs. 29.25 ± 8.32 mmol/L, *p* < 0.001) values in the LD group increased significantly. There was no significant difference in PH and ${\text{HCO}}_{3}^{-}$ values either before or after treatment in the LF group, or between the LF and LD groups before treatment. Meanwhile, there was no significant difference in the incidence of severe acidosis and alkalosis, not only in the LD and LF groups but also in the LD group or the LF group before and after the treatment (Table 2).

In the LF group, the average serum sodium decreased significantly after treatment compared with before treatment (139.84 ± 8.28 vs. 137.65 ± 5.74 mmol/L, *p* = 0.01), but both maintain in the normal range; while in the LD group, serum sodium increased significantly after treatment (134.93 ± 6.16 vs. 138.00 ± 5.16 mmol/L, *p* = 0.01). Before treatment, the LF group had a higher incidence of hypernatremia (7.2 vs. 0%, *p* < 0.001) and a lower incidence of hyponatremia (24.1 vs. 51.5%, *p* < 0.001) than the LF group. There was no significant difference in the incidence of hypernatremia and hyponatremia before and after treatment either in the LF group or the LD group.

### CRRT filter lifespan

**Figure 2:**
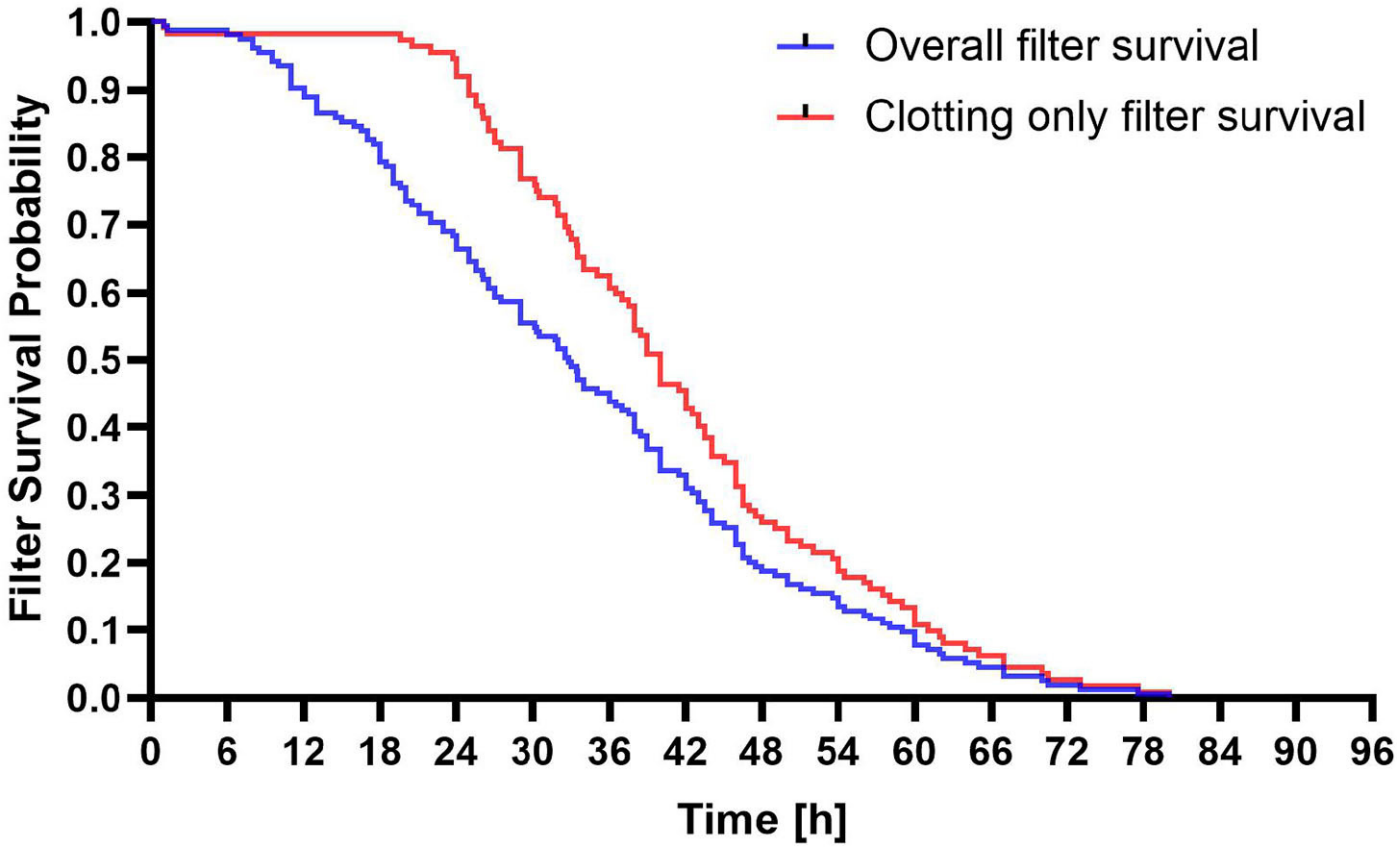
Survival probability of filters of regional citrate anticoagulation and continuous renal replacement therapy (RCA-CRRT) circuits. Blue line: All filter discontinuations. Red line: Clotting only related filter discontinuations.

The average filter lifespan of 151 RCA-CRRT sessions was 34.33 ± 17.1 h. The relationship between filter lifespan and clotting is shown in Figure 2. There was no significant difference in the average filter lifespan between the LF and LD groups (35.63 ± 17.41 h vs. 31.94 ± 16.92 h, *p* = 0.18).

### Filter clotting and active bleeding

Among the 75 children, there was no significant difference in the incidence of bleeding between the LF and LD groups (9.5 vs. 9.1%, *p* = 0.95). A total of 43 in 151 sessions (28.5%) filter lifespan < 24 h occurred. There was no significant difference in the incidence of filter lifespan < 24 h between the LF and LD groups (25.3 vs. 32.4%, *p* = 0.68; Table 1).

### Changes in coagulation indicators after treatment

PT and APTT in the LF group were significantly higher than those in the LD group both before and after treatment. But, there was no significant difference in PT and APTT before and after treatment in either the LF group or the LD group (Table 3).

**Table 3:** Biochemical and coagulation indicators of LF group and LD group before and after treatment.

**Item**	**LF group (*****n*** = **83)**		**LD group (*****n*** = **68)**		***p*-Value**
	**Before treatment**	**After treatment**	**Before treatment**	**After treatment**	
TB (μmol/L)	102.69 ± 10.93	116.10 ± 12.49	14.78 ± 4.62	20.04 ± 4.78	P^F*^= 0.06, P^D*^= 0.10 P^FD#^ < 0.001, P^FD&^ < 0.001
**DB (μmol/L)**	44.28 ± 7.83	47.48 ± 8.08	5.44 ± 3.26	6.07 ± 2.87	P^F*^= 0.48, P^D*^= 0.71 P^FD#^ < 0.001, P^FD&^ < 0.001
ALT (U/L)	1092.60 ± 262.64	509.14 ± 91.65	313.35 ± 100.97	233.48 ± 54.49	P^F*^= 0.07, P^D*^= 0.14 P^FD#^ = 0.07, P^FD&^ = 0.05
AST (U/L)	1675.74 ± 448.55	771.78 ± 212.81	342.70 ± 116.42	242.45 ± 57.72	P^F*^= 0.03, P^D*^= 0.12 P^FD#^ = 0.01, P^FD&^ = 0.03
GGT (U/L)	61.60 ± 94.14	34.99 ± 66.93	35.30 ± 38.73	14.94 ± 16.65	P^F*^ < 0.001, P^D*^ < 0.001 P^FD#^ = 0.04, P^FD&^ = 0.02
Creatinine (μmol/L)	128.46 ± 18.19	62.18 ± 4.61	226.68 ± 33.02	100.04 ± 11.25	P^F*^ < 0.001, P^D*^ < 0.001 P^FD#^ < 0.001, P^FD&^ < 0.001
Serum ammonia (μmol/L)	82.26 ± 7.76	47.97 ± 6.28	40.18 ± 8.40	28.13 ± 5.57	P^F*^ < 0.001, P^D*^= 0.24 P^FD#^ = 0.02, P^FD&^ = 0.12
Lactic acid (μmol/L)	10.29 ± 5.09	2.50 ± 0.36	4.93 ± 1.97	1.76 ± 0.58	P^F*^= 0.13, P^D*^= 0.09 P^FD#^ = 0.35, P^FD&^ = 0.14
APTT (s)	63.13 ± 4.99	57.61 ± 4.96	46.75 ± 5.12	36.77 ± 2.52	P^F*^= 0.34, P^D*^= 0.03 P^FD#^ < 0.001, P^FD&^ < 0.001
PT (s)	30.80 ± 4.21	34.22 ± 5.20	14.99 ± 0.51	14.19 ± 0.75	P^F*^= 0.45, P^D*^= 0.24 P^FD#^ < 0.001, P^FD&^ = 0.01
TCa (mmol/L)	2.39 ± 0.49	2.69 ± 0.61	2.10 ± 0.41	2.51 ± 0.31	P^F*^ < 0.001, P^D*^ < 0.001 P^FD#^ < 0.001, P^FD&^ = 0.05
iCa in vivo (mmol/L)	0.99 ± 0.19	1.11 ± 0.17	1.05 ± 0.16	1.16 ± 0.15	P^F*^ < 0.001, P^D*^ < 0.001 P^FD#^ = 0.10, P^FD&^ = 0.19

LF, liver failure; LD, liver dysfunction; TB, total bilirubin; DB, direct or conjugated bilirubin; AST, aspartate aminotransferase; ALT, alanine aminotransferase; GGT, glutamyl transpeptidase; APTT, activated partial thromboplastin time; PT, prothrombin time; TCa, total calcium; iCa *in vivo*, ionized calcium *in vivo*; P^F*^ is the comparison of the LF group between before and after treatment, P^D*^ is the comparison of the LD group between before and after treatment, P^FD#^ is the comparison before treatment between the LF and LD groups, and P^FD&^ is the comparison after treatment between the LF and LD groups.

### Changes in biochemical indicators after treatment

The LF group had significantly higher total bilirubin and direct bilirubin than the LD group both before and after treatment. ALT, AST, and GGT decreased after treatment in LD and LF groups compared with before treatment, but only GGT with a significant decrease in the LD and LF groups and AST with a significant decrease in the LF group. After treatment, the levels of lactic acid, serum ammonia, and creatinine decreased significantly in either the LF group or LD group (Table 3).

### Final RCA protocol

There was no significant difference in QB (3.71 ± 0.99 vs. 3.64 ± 1.07, *p* = 0.54), QCi (5.62 ± 1.47 vs. 6.19 ± 1.82, *p* = 0.23) and Qd + Qf (95.24 ± 46.75 vs. 92.73 ± 56.25, *p* = 0.75), and QCa (0.60 ± 0.61 vs. 0.47 ± 0.50, *p* = 0.17) between LF group and LD group at the end of treatment. The QSB was significantly higher in the LF group than that in the LD group (0.78 ± 0.95 vs. 0.43 ± 0.44, *p* < 0.001; Table 4).

**Table 4:** Comparison of RCA-CRRT parameters at the beginning and end of the LD and LF groups.

	**LF group (*****n*** = **83)**		**LD group (*****n*** = **68)**		***p*-Value**
	**Initial parameters**	**Final parameters**	**Initial parameters**	**Final parameters**	
QB (ml/kg/min)	5	3.71 ± 0.99	5	3.64 ± 1.07	0.54
QCi (ml/kg/h)	9	5.62 ± 1.47	9	6.19 ± 1.82	0.23
QCa (ml/kg/h)	0.5	0.60 ± 0.61	0.5	0.47 ± 0.50	0.17
Qd + Qf (ml/kg/h)	60	95.24 ± 46.75	60	92.73 ± 56.25	0.75
QSB (ml/kg/h)	0.6	0.78 ± 0.95	0.6	0.43 ± 0.44	< 0.001
QCi/QB	1.8	1.51	1.8	1.70	
QCa/QB	0.1	0.16	0.1	0.13	
QSB/(Qd+Qf)	0.01	0.0082	0.01	0.0046	

LF, liver failure; LD, liver dysfunction; QB, blood flow rate; QCi, the flow rate of 4% sodium citrate; QCa, the flow rate of 10% calcium gluconate; Qd, the flow rate of dialysate; Qf, the flow rate of replacement fluid; QSB, the flow rate of sodium bicarbonate.

## Discussion

In this study, we found that children with different degrees of liver damage can be effectively treated with RCA-CRRT. When RCA-CRRT was performed in children with LF, the risks of CA and hypocalcemia were significantly higher than that of children with LD, but both risks could be significantly reduced by adjusting the RCA-CRRT regimen properly. There was no significant difference between children with LF and those with LD in the complications of acid–base imbalance and sodium disorder.

RCA is especially suitable for CRRT in patients with high bleeding risk due to its full anticoagulation *in vitro* and almost no effect *in vivo*. Critically ill children are often complicated with LD or even LF, which increases the risk of coagulation disorders and bleeding. Since citrate is mainly metabolized in the liver, LF has been listed as a contraindication for RCA-CRRT. However, with the continuous research of RCA-CRRT, more and more scholars believe that it can be used in patients with LF (6, 17–21). However, RCA-CRRT has been rarely reported in children with LF (11, 12, 22). Due to the unfamiliarity with RCA-CRRT, many pediatricians are concerned about using RCA-CRRT in children with LD, let alone children with LF.

RCA can achieve effective anticoagulation for CRRT in children with LD or LF without additional bleeding risk. Although seven children were complicated with bleeding during the RCA-CRRT process, all of them were considered to be related to their own diseases rather than caused by RCA-CRRT. The incidence of filter lifespan < 24 h in this group was about 28.5%, which was similar to the findings of Brophy et al. (27%) (11).

The incidence of CA in the LF group was significantly higher than that in the LD group, and the incidence of CA in the two groups could be significantly reduced by adjusting the RCA-protocol properly. Citrate accumulation is a major complication of RCA-CRRT and a major concern for most clinicians. In our research, the incidences of TCA (40.5 vs. 15.2%) and PCA (8.8 vs. 1.4%) in the LF group were significantly higher than those in the LD group, which were significantly lower than those of Rodriguez et al. (22) (The incidence of TCA was 70%, and the incidence of PCA was 42.9%). This suggests that the liver's ability to metabolize citric acid is related to the degree of liver injury. In addition, children with LF are less able to metabolize citric acid than those with LD, rather than completely losing the ability to metabolize citric acid.

Although CA is the main complication of RCA, its main side effect is hypocalcemia and hypocalcemia-related complications because citrate itself is not toxic. Children with LF often have hypocalcemia even without CRRT. Meanwhile, hypocalcemia is one of the most serious complications of citrate anticoagulation, because severe hypocalcemia can cause hypotension, convulsions, arrhythmias, and respiratory muscle weakness. In this study, the incidence of hypocalcemia before treatment in the LF group was as high as 34.9%. However, the incidence of hypocalcemia-related hypotension was only 5.9%−6.0%, and the incidence of hypocalcemia-related convulsions was only 1.5%−2.4%. The incidence of hypocalcemia in the LF group before and after treatment was significantly higher than that in the LD group, and the incidence of hypocalcemia in the two groups could be significantly reduced by adjusting the RCA-protocol properly, too.

In addition to CA and hypocalcemia complications, RCA also mainly leads to other metabolic complications such as acid–base imbalance and sodium disorder. After treatment, there were no significant differences in the incidence of hypercalcemia, severe acid and alkalosis, hypernatremia, and hypokalemia between the two groups. This suggests that the complications of acid–base and sodium disorders related to RCA-CRRT treatment in our research were not affected by the degree of liver injury.

RCA can maintain the effective anticoagulation requirement of CRRT in children with liver injury. The principle of RCA is that citrate can chelate ionized calcium, which can reduce the concentration of ionized calcium to block the coagulation process. It has been found that when the ionized calcium concentration is lower than 0.33 mmol/L, the blood will not clot, while when the ionized calcium concentration is higher than 0.56 mmol/L, the blood will clot normally (23). Therefore, citrate is currently the most ideal anticoagulation in CRRT that can maintain adequate anticoagulation *in vitro* and normal blood coagulation *in vivo*. In our study, the average filter lifespan of the LF group and LD group (35.63 ± 17.41 h vs. 31.94 ± 16.92 h) was between most other studies (from 29 to 66 h) (17, 22, 24, 25), but was still relatively short.

It may be related that more attention was paid to the risk of CA [the risk of CA was lower in this study than that of Rodriguez et al. (22)], and the average ionic calcium *in vitro* level was relatively high. The average concentration of ionic calcium *in vitro* of the LF group and LD group was 0.36 ± 0.09 vs. 0.35 ± 0.08 mmol/L, and the rate of ionic calcium concentration below 0.4 mmol/L was 68.25 vs. 70.98%.

When RCA-CRRT is used in children with liver injury, the anticoagulant effect is mainly related to the concentration of ionized calcium *in vitro*, but the self-coagulation function may also be an important factor. In addition, the average filter time in the LF group was slightly longer than that in the LD group in our study, without significant differences in the concentration of ionic calcium *in vitro* and the rate of ionic calcium concentration below 0.4 mmol/L during CRRT between the two groups, while APTT and PT in the LF group were significantly longer than that in the LD group. It is suggested that the lifter lifespan of RCA-CRRT is mainly related to the concentration of ionic calcium *in vitro*, and may also be related to the coagulation function status of the child.

After treatment, blood ammonia and lactic acid were reduced to normal levels, and creatinine was also significantly reduced compared to before treatment. It is suggested that the adjustment scheme in our study can still meet the therapeutic needs of CRRT.

The RCA-CRRT program is affected by multiple factors, such as the degree of liver injury and its own disease. Through continuous testing and proper adjustment of the parameters, RCA-CRRT-related complications can be significantly reduced and the safety and efficacy of RCA-CRRT can be increased. When citrate accumulation occurs, we often directly reduced citrate consumption by replacing calcium-free replacement fluid or slowing blood flow to reduce citrate dosage or increasing replacement/dialysis volume to increase citrate clearance, thereby reducing CA (26). The protocol after adjustment in children with LF was about QB = 3.7 ml/kg/min, QCi (ml/h) = 1.5 QB (ml/min), Qd + Qf = 95 ml/kg/h, QSB (ml/h) = 0.008 (Qf + Qd), and QCa (ml/h) = 0.16 QB (ml/min). The incidence of CA decreased significantly from 40.5 to 8.8%, while the incidence of severe hypocalcemia was only 1.86%. It is suggested that the risk of CA can be effectively reduced by replacing calcium-free replacement fluid, increasing replacement/dialysis volume, and slowing down blood flow rate (accompanied by lower citrate) in children with LF undergoing RCA-CRRT. At the same time, severe hypocalcemia can be effectively controlled by reducing the risk of CA and increasing the dose of calcium. After the treatment, the average level of acid–base balance and electrolyte was well maintained: pH (7.40 ± 0.14), ${\text{HCO}}_{3}^{-}$ (24.37 ± 7.79 mmol/L), Na^+^ (137.65 ± 5.74 mmol/L), and Ca^2+^ (1.11 ± 0.17 mmol/L).

## Limitation

Limitations of our study include the retrospective nature of data collection. The number of included patients is quite small, which may result in bias. Furthermore, not all the laboratory examinations were done and available to all the patients at all time points desired for this evaluation.

## Conclusion

The risk of CA in RCA-CRRT in children with LF is higher than that in patients with LD. The incidence of CA can be significantly reduced and RCA-related acid–base imbalance and electrolyte disorder complications can be effectively controlled through proper regulation of RCA parameters.

## Data availability statement

The data analyzed in this study is not readily available due to patient privacy concerns. Requests to access the de-identified datasets should be directed to the corresponding author.

## Ethics statement

This study has been reviewed and approved by the Ethics Committee of the Children's Hospital of Chongqing Medical University, with ethical review number: (2020) NLS (Y) No. 131. Written informed consent from the patients/participants legal guardian/next of kin was not required to participate in this study in accordance with the national legislation and the institutional requirements.

## Author contributions

FH: data collection. YS: article writing. KB: topic design, scheme formulation, and overall quality control. CL: expert guidance, opinions, and suggestions. All authors contributed to the article and approved the submitted version.

## Funding

This work was supported by the Program for Youth Innovation in Future Medicine from Chongqing Medical University: Basic and Clinical Study of Critical Illness in Children (2021-W0111; Dang Hongxing).

## Conflict of interest

The authors declare that the research was conducted in the absence of any commercial or financial relationships that could be construed as a potential conflict of interest.

## Publisher's note

All claims expressed in this article are solely those of the authors and do not necessarily represent those of their affiliated organizations, or those of the publisher, the editors and the reviewers. Any product that may be evaluated in this article, or claim that may be made by its manufacturer, is not guaranteed or endorsed by the publisher.
